# 3-Hydr­oxy-1,2-dimethoxy­anthraquinone

**DOI:** 10.1107/S1600536809021266

**Published:** 2009-06-06

**Authors:** Yong-Jun Xu, Xiao-Xi Yang, Hong-Bin Zhao

**Affiliations:** a College of Chemistry and Environmental Engineering, Dongguan University of Technology, Dongguan 523808, People’s Republic of China

## Abstract

The title compound, C_16_H_12_O_5_, was isolated from *Morinda officinalis* How. The anthraquinone ring system is almost planar, the dihedral angle between the two benzene rings being 1.12 (4)°. In the crystal structure, O—H⋯O and C—H⋯O hydrogen bonds link the mol­eculesin the crystallographic *a*-axis direction. Weak π–π stacking inter­actions [centroid–centroid distance between symmetry-related benzene rings of 3.699 (4) Å] are also present.

## Related literature

For the biological properties of anthraquinone derivatives, see: Kim *et al.* (2005 ▶) and of the title compound, see: Ali *et al.* (2000 ▶); Jia *et al.* (2007 ▶); Wu *et al.* (2003 ▶). For related structures, see: Ng *et al.* (2005 ▶); Boonnak *et al.* (2005 ▶). For the structure of another compound isolated from *Morinda officinalis* How., see: Liu & Jiao (2009 ▶). For reference structural data, see: Allen *et al.* (1987 ▶).
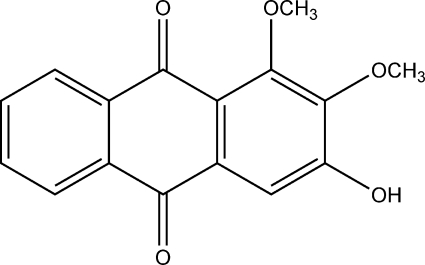


## Experimental

### 

#### Crystal data

C_16_H_12_O_5_
*M* *_r_* = 284.26
Triclinic, 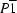

*a* = 7.4087 (17) Å
*b* = 8.0387 (18) Å
*c* = 11.802 (3) Å
α = 95.386 (3)°
β = 92.357 (3)°
γ = 115.712 (2)°
*V* = 627.9 (3) Å^3^
*Z* = 2
Mo *K*α radiation
μ = 0.11 mm^−1^
*T* = 293 K
0.30 × 0.20 × 0.20 mm

#### Data collection

Bruker APEXII area-detector diffractometer
Absorption correction: multi-scan (*SADABS*; Sheldrick, 1996 ▶) *T*_min_ = 0.967, *T*_max_ = 0.978
3200 measured reflections
2182 independent reflections
1639 reflections with *I* > 2σ(*I*)
*R*_int_ = 0.013

#### Refinement

*R*[*F* ^2^ > 2σ(*F* ^2^)] = 0.044
*wR*(*F* ^2^) = 0.136
*S* = 1.06
2182 reflections
210 parameters
H atoms treated by a mixture of independent and constrained refinement
Δρ_max_ = 0.25 e Å^−3^
Δρ_min_ = −0.17 e Å^−3^

### 

Data collection: *APEX2* (Bruker, 2004 ▶); cell refinement: *SAINT* (Bruker, 2004 ▶); data reduction: *SAINT* (Bruker, 2004 ▶); program(s) used to solve structure: *SHELXS97* (Sheldrick, 2008 ▶); program(s) used to refine structure: *SHELXL97* (Sheldrick, 2008 ▶); molecular graphics: *XP* in *SHELXTL* (Sheldrick, 2008 ▶); software used to prepare material for publication: *SHELXL97*.

## Supplementary Material

Crystal structure: contains datablocks I, global. DOI: 10.1107/S1600536809021266/pk2164sup1.cif

Structure factors: contains datablocks I. DOI: 10.1107/S1600536809021266/pk2164Isup2.hkl

Additional supplementary materials:  crystallographic information; 3D view; checkCIF report

## Figures and Tables

**Table 1:** Hydrogen-bond geometry (Å, °)

*D*—H⋯*A*	*D*—H	H⋯*A*	*D*⋯*A*	*D*—H⋯*A*
C25—H5⋯O1^i^	0.97 (3)	2.57 (3)	3.256 (2)	128 (2)
O8—H6⋯O1^i^	0.88 (3)	1.91 (3)	2.781 (2)	168 (3)

Symmetry code: (i) 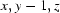.

**Fig. 1.**
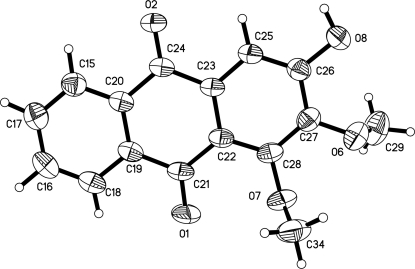
The molecular structure showing the atomic-numbering scheme and displacement ellipsoids drawn at the 30% probability level.

**Fig. 2.**
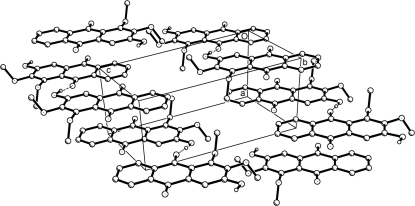
The molecular packing showing the hydrogen bonding interactions as broken lines.

## supplementary crystallographic information

### Comment

Anthraquinone derivatives extracted from the roots of Morinda officinalis How. (most common familiar name in China: Bajitian) have been used to support the entire body treating a wide range of symptoms, including poor digestion, high blood pressure and immune deficiencies in China since ancient times. Recent studies have demonstrated that they have multiple pharmacological actions such as anti-HIV, anti-inflammatory, antinociceptive, antimicrobial, antioxidant, antihepatotoxic and antimutagenic activities (Kim et al., 2005). One component found in Morinda officinalis How., 1,2-dimethoxy-3-hydroxyanthraquinone, exhibits a variety of potent biological effects such as antiviral and antimicrobial activities (Ali et al., 2000), antioxidant activity (Jia et al., 2007) and cyototoxic activity (Wu et al., 2003). We report here the structure of the title compound.

In the title compound (Fig. 1), the C-C bond lengths show normal values (Allen et al., 1987), and the C-O and C=O bond lengths are comparable to those observed in similar structures (Ng et al., 2005; Boonnak et al., 2005). The anthraquinone ring system is substantially planar, the dihedral angle between the two benzene rings being 1.12 (4)°. The molecules are self-assembled by C—H···O and O—H···.O hydrogen bonding interactions (Table 1) into a supramolecular network. The crystal structure is further stabilized by weak π-π interactions along the a axis (Fig. 2) between the anthraquinone ring systems of the stacked molecules. The centroid-to-centriod distances between related benzene rings of the stacked molecules is 3.699 (4)Å, thus indicating weak π-π contacts.

### Experimental

The roots of *Morinda officinalis How*. (1000 g) were shattered to powder (about 30 mesh) and extracted with 85% ethanol (4000 ml) for 2 h with stirring. The extraction procedure was repeated three times. The extracts were combined and evaporated to dryness under reduced pressure at 333 K, the residue was redissolved in water (800 ml). Then the enriched extracts were extracted with chloroform three times (800 ml each), the chlorofrom solutions were combined and evaporated to dryness under reduced pressure at 333 K, 6.80 g crude extracts were obtained. The crude extracts were separated with n-hexane-ethyl acetate-methanol-water (6 : 4 : 5 : 5, v/v) using high-speed counter-current chromatography (HSCCC) to obtain 1,2-dimethoxy-3-hydroxyanthraquinone (yield 20.3 mg). Single crystals suitable for X-ray analysis were obtained by slow evaporation of a methanol solution.

### Refinement

Methyl H atoms were placed at calculated positions and treated as riding on the parent C atoms with C—H = 0.96 °H and *U*_iso_(H) = 1.2*U*_eq_(C).  Coordinates of all other hydrogens were refined but their *U*_iso_ values were fixed at 0.105 Å^2^.

### Crystal data

**Table:**

C_16_H_12_O_5_	*Z* = 2
*M**_r_* = 284.26	*F*(000) = 296.0
Triclinic, *P*1	*D*_x_ = 1.503 Mg m^−^^3^
Hall symbol: -P 1	Mo *K*α radiation, λ = 0.71073 Å
*a* = 7.4087 (17) Å	Cell parameters from 1305 reflections
*b* = 8.0387 (18) Å	θ = 3.1–27.3°
*c* = 11.802 (3) Å	µ = 0.11 mm^−^^1^
α = 95.386 (3)°	*T* = 293 K
β = 92.357 (3)°	Block, yellow
γ = 115.712 (2)°	0.30 × 0.20 × 0.20 mm
*V* = 627.9 (3) Å^3^	

### Data collection

**Table:**

Bruker APEXII area-detector diffractometer	2182 independent reflections
Radiation source: fine-focus sealed tube	1639 reflections with *I* > 2σ(*I*)
graphite	*R*_int_ = 0.013
φ and ω scans	θ_max_ = 25.0°, θ_min_ = 2.8°
Absorption correction: multi-scan (*SADABS*; Sheldrick, 1996)	*h* = −7→8
*T*_min_ = 0.967, *T*_max_ = 0.978	*k* = −9→9
3200 measured reflections	*l* = −14→11

### Refinement

**Table:**

Refinement on *F*^2^	Primary atom site location: structure-invariant direct methods
Least-squares matrix: full	Secondary atom site location: difference Fourier map
*R*[*F*^2^ > 2σ(*F*^2^)] = 0.044	Hydrogen site location: inferred from neighbouring sites
*wR*(*F*^2^) = 0.136	H atoms treated by a mixture of independent and constrained refinement
*S* = 1.06	*w* = 1/[σ^2^(*F*_o_^2^) + (0.0736*P*)^2^ + 0.1017*P*] where *P* = (*F*_o_^2^ + 2*F*_c_^2^)/3
2182 reflections	(Δ/σ)_max_ < 0.001
210 parameters	Δρ_max_ = 0.25 e Å^−^^3^
0 restraints	Δρ_min_ = −0.17 e Å^−^^3^

### Special details

**Table:**

Geometry. All e.s.d.'s (except the e.s.d. in the dihedral angle between two l.s. planes) are estimated using the full covariance matrix. The cell e.s.d.'s are taken into account individually in the estimation of e.s.d.'s in distances, angles and torsion angles; correlations between e.s.d.'s in cell parameters are only used when they are defined by crystal symmetry. An approximate (isotropic) treatment of cell e.s.d.'s is used for estimating e.s.d.'s involving l.s. planes.
Refinement. Refinement of *F*^2^ against ALL reflections. The weighted *R*-factor *wR* and goodness of fit *S* are based on *F*^2^, conventional *R*-factors *R* are based on *F*, with *F* set to zero for negative *F*^2^. The threshold expression of *F*^2^ > σ(*F*^2^) is used only for calculating *R*-factors(gt) *etc*. and is not relevant to the choice of reflections for refinement. *R*-factors based on *F*^2^ are statistically about twice as large as those based on *F*, and *R*- factors based on ALL data will be even larger.

### Fractional atomic coordinates and isotropic or equivalent isotropic displacement parameters (Å^2^)

**Table:**

	*x*	*y*	*z*	*U*_iso_*/*U*_eq_	
O1	0.2605 (2)	0.79647 (18)	0.14638 (13)	0.0605 (4)	
O2	0.2359 (2)	0.18299 (17)	−0.09353 (11)	0.0560 (4)	
O6	0.2197 (2)	0.3418 (2)	0.43860 (11)	0.0597 (4)	
O7	0.2427 (2)	0.64028 (18)	0.34133 (12)	0.0547 (4)	
O8	0.1946 (2)	0.03307 (19)	0.30888 (12)	0.0582 (4)	
C15	0.2626 (3)	0.4756 (3)	−0.21242 (16)	0.0463 (5)	
C16	0.2985 (3)	0.7870 (3)	−0.20248 (19)	0.0550 (5)	
C17	0.2839 (3)	0.6275 (3)	−0.26601 (19)	0.0546 (5)	
C18	0.2898 (3)	0.7953 (3)	−0.08620 (19)	0.0474 (5)	
C19	0.2686 (2)	0.6434 (2)	−0.03041 (15)	0.0379 (4)	
C20	0.2551 (2)	0.4817 (2)	−0.09503 (15)	0.0372 (4)	
C21	0.2574 (3)	0.6545 (2)	0.09545 (16)	0.0403 (4)	
C22	0.2422 (2)	0.4946 (2)	0.15295 (15)	0.0368 (4)	
C23	0.2284 (2)	0.3315 (2)	0.08725 (14)	0.0350 (4)	
C24	0.2378 (2)	0.3203 (2)	−0.03826 (15)	0.0377 (4)	
C25	0.2095 (3)	0.1777 (2)	0.13812 (16)	0.0399 (4)	
C26	0.2058 (3)	0.1783 (2)	0.25489 (15)	0.0425 (4)	
C27	0.2176 (3)	0.3354 (3)	0.32218 (15)	0.0440 (5)	
C28	0.2364 (3)	0.4919 (2)	0.27130 (15)	0.0405 (4)	
C29	0.0304 (4)	0.2459 (4)	0.4794 (2)	0.0804 (8)	
H29A	−0.0439	0.3180	0.4754	0.097*	
H29B	0.0483	0.2264	0.5573	0.097*	
H29C	−0.0422	0.1278	0.4333	0.097*	
C34	0.4369 (4)	0.7632 (3)	0.3940 (2)	0.0703 (7)	
H34A	0.4862	0.6969	0.4403	0.084*	
H34B	0.4295	0.8636	0.4412	0.084*	
H34C	0.5266	0.8121	0.3363	0.084*	
H1	0.249 (4)	0.360 (4)	−0.255 (2)	0.105*	
H4	0.298 (5)	0.897 (4)	−0.044 (3)	0.105*	
H3	0.323 (4)	0.897 (4)	−0.239 (2)	0.105*	
H2	0.292 (4)	0.616 (4)	−0.345 (3)	0.105*	
H5	0.198 (4)	0.069 (4)	0.089 (3)	0.105*	
H6	0.199 (5)	−0.044 (4)	0.253 (3)	0.105*	

### Atomic displacement parameters (Å^2^)

**Table:**

	*U*^11^	*U*^22^	*U*^33^	*U*^12^	*U*^13^	*U*^23^
O1	0.0852 (11)	0.0373 (7)	0.0687 (9)	0.0365 (7)	0.0148 (8)	−0.0003 (6)
O2	0.0889 (11)	0.0383 (7)	0.0479 (8)	0.0358 (7)	0.0051 (7)	−0.0014 (6)
O6	0.0681 (10)	0.0683 (10)	0.0419 (8)	0.0301 (8)	0.0044 (7)	0.0023 (7)
O7	0.0608 (9)	0.0478 (8)	0.0564 (8)	0.0291 (7)	0.0023 (7)	−0.0142 (6)
O8	0.0884 (11)	0.0506 (9)	0.0507 (8)	0.0423 (8)	0.0151 (7)	0.0147 (6)
C15	0.0462 (11)	0.0455 (11)	0.0489 (11)	0.0220 (9)	0.0019 (8)	0.0054 (8)
C16	0.0524 (12)	0.0451 (12)	0.0689 (14)	0.0202 (10)	0.0041 (10)	0.0208 (10)
C17	0.0569 (13)	0.0551 (12)	0.0534 (12)	0.0247 (10)	0.0048 (10)	0.0143 (10)
C18	0.0420 (11)	0.0346 (10)	0.0673 (13)	0.0183 (9)	0.0037 (9)	0.0076 (9)
C19	0.0293 (9)	0.0313 (9)	0.0540 (11)	0.0144 (7)	0.0026 (7)	0.0046 (8)
C20	0.0295 (9)	0.0320 (9)	0.0501 (11)	0.0140 (7)	0.0020 (7)	0.0032 (7)
C21	0.0349 (9)	0.0291 (9)	0.0575 (11)	0.0161 (8)	0.0050 (8)	−0.0014 (8)
C22	0.0312 (9)	0.0307 (9)	0.0491 (11)	0.0152 (7)	0.0045 (7)	0.0000 (7)
C23	0.0310 (9)	0.0276 (9)	0.0460 (10)	0.0135 (7)	0.0034 (7)	−0.0005 (7)
C24	0.0347 (9)	0.0289 (9)	0.0480 (10)	0.0140 (7)	0.0012 (7)	−0.0004 (7)
C25	0.0419 (10)	0.0316 (9)	0.0476 (11)	0.0179 (8)	0.0055 (8)	0.0020 (7)
C26	0.0451 (11)	0.0388 (10)	0.0472 (11)	0.0213 (9)	0.0067 (8)	0.0071 (8)
C27	0.0439 (11)	0.0496 (11)	0.0400 (10)	0.0228 (9)	0.0043 (8)	0.0008 (8)
C28	0.0363 (10)	0.0386 (10)	0.0470 (10)	0.0191 (8)	0.0027 (7)	−0.0060 (8)
C29	0.0866 (18)	0.102 (2)	0.0514 (13)	0.0379 (16)	0.0221 (12)	0.0158 (13)
C34	0.0775 (16)	0.0484 (12)	0.0745 (15)	0.0243 (12)	−0.0103 (12)	−0.0172 (11)

### Geometric parameters (Å, °)

**Table:**

O1—C21	1.230 (2)	C19—C21	1.488 (3)
O2—C24	1.222 (2)	C20—C24	1.475 (2)
O6—C27	1.369 (2)	C21—C22	1.473 (3)
O6—C29	1.407 (3)	C22—C28	1.401 (3)
O7—C28	1.368 (2)	C22—C23	1.421 (2)
O7—C34	1.418 (3)	C23—C25	1.380 (3)
O8—C26	1.355 (2)	C23—C24	1.482 (3)
O8—H6	0.88 (3)	C25—C26	1.379 (3)
C15—C17	1.379 (3)	C25—H5	0.97 (3)
C15—C20	1.386 (3)	C26—C27	1.394 (3)
C15—H1	0.98 (3)	C27—C28	1.400 (3)
C16—C18	1.373 (3)	C29—H29A	0.9600
C16—C17	1.382 (3)	C29—H29B	0.9600
C16—H3	0.97 (3)	C29—H29C	0.9600
C17—H2	0.94 (3)	C34—H34A	0.9600
C18—C19	1.394 (3)	C34—H34B	0.9600
C18—H4	0.90 (3)	C34—H34C	0.9600
C19—C20	1.405 (2)		
			
C27—O6—C29	114.93 (16)	C22—C23—C24	121.57 (16)
C28—O7—C34	114.31 (15)	O2—C24—C20	120.49 (16)
C26—O8—H6	102 (2)	O2—C24—C23	121.23 (16)
C17—C15—C20	120.31 (19)	C20—C24—C23	118.26 (15)
C17—C15—H1	122.3 (18)	C26—C25—C23	120.99 (16)
C20—C15—H1	117.3 (18)	C26—C25—H5	121.2 (18)
C18—C16—C17	120.59 (19)	C23—C25—H5	117.8 (18)
C18—C16—H3	119.1 (17)	O8—C26—C25	123.02 (16)
C17—C16—H3	120.2 (17)	O8—C26—C27	117.56 (16)
C15—C17—C16	120.0 (2)	C25—C26—C27	119.41 (17)
C15—C17—H2	116.1 (19)	O6—C27—C26	120.69 (17)
C16—C17—H2	123.9 (19)	O6—C27—C28	119.27 (16)
C16—C18—C19	120.33 (19)	C26—C27—C28	119.98 (17)
C16—C18—H4	122 (2)	O7—C28—C27	117.34 (16)
C19—C18—H4	118 (2)	O7—C28—C22	121.20 (17)
C18—C19—C20	119.00 (18)	C27—C28—C22	121.44 (15)
C18—C19—C21	119.49 (16)	O6—C29—H29A	109.5
C20—C19—C21	121.50 (16)	O6—C29—H29B	109.5
C15—C20—C19	119.80 (16)	H29A—C29—H29B	109.5
C15—C20—C24	119.84 (16)	O6—C29—H29C	109.5
C19—C20—C24	120.34 (16)	H29A—C29—H29C	109.5
O1—C21—C22	123.18 (18)	H29B—C29—H29C	109.5
O1—C21—C19	118.37 (17)	O7—C34—H34A	109.5
C22—C21—C19	118.45 (14)	O7—C34—H34B	109.5
C28—C22—C23	116.89 (16)	H34A—C34—H34B	109.5
C28—C22—C21	123.33 (15)	O7—C34—H34C	109.5
C23—C22—C21	119.77 (16)	H34A—C34—H34C	109.5
C25—C23—C22	121.27 (17)	H34B—C34—H34C	109.5
C25—C23—C24	117.15 (15)		
			
C20—C15—C17—C16	0.1 (3)	C19—C20—C24—C23	2.0 (2)
C18—C16—C17—C15	−0.6 (3)	C25—C23—C24—O2	−2.5 (3)
C17—C16—C18—C19	0.7 (3)	C22—C23—C24—O2	176.29 (16)
C16—C18—C19—C20	−0.4 (3)	C25—C23—C24—C20	179.16 (14)
C16—C18—C19—C21	−179.35 (16)	C22—C23—C24—C20	−2.1 (2)
C17—C15—C20—C19	0.2 (3)	C22—C23—C25—C26	−0.8 (3)
C17—C15—C20—C24	−178.33 (16)	C24—C23—C25—C26	178.01 (15)
C18—C19—C20—C15	−0.1 (3)	C23—C25—C26—O8	−177.61 (16)
C21—C19—C20—C15	178.84 (15)	C23—C25—C26—C27	1.2 (3)
C18—C19—C20—C24	178.43 (15)	C29—O6—C27—C26	−78.9 (2)
C21—C19—C20—C24	−2.6 (2)	C29—O6—C27—C28	103.9 (2)
C18—C19—C21—O1	2.4 (3)	O8—C26—C27—O6	0.7 (3)
C20—C19—C21—O1	−176.58 (15)	C25—C26—C27—O6	−178.22 (16)
C18—C19—C21—C22	−177.85 (15)	O8—C26—C27—C28	177.81 (16)
C20—C19—C21—C22	3.2 (2)	C25—C26—C27—C28	−1.1 (3)
O1—C21—C22—C28	−2.1 (3)	C34—O7—C28—C27	85.1 (2)
C19—C21—C22—C28	178.18 (15)	C34—O7—C28—C22	−96.8 (2)
O1—C21—C22—C23	176.54 (16)	O6—C27—C28—O7	−4.2 (3)
C19—C21—C22—C23	−3.2 (2)	C26—C27—C28—O7	178.56 (15)
C28—C22—C23—C25	0.2 (3)	O6—C27—C28—C22	177.70 (15)
C21—C22—C23—C25	−178.54 (15)	C26—C27—C28—C22	0.5 (3)
C28—C22—C23—C24	−178.54 (14)	C23—C22—C28—O7	−178.03 (14)
C21—C22—C23—C24	2.8 (2)	C21—C22—C28—O7	0.6 (3)
C15—C20—C24—O2	2.2 (3)	C23—C22—C28—C27	0.0 (3)
C19—C20—C24—O2	−176.41 (16)	C21—C22—C28—C27	178.61 (15)
C15—C20—C24—C23	−179.46 (15)		

### Hydrogen-bond geometry (Å, °)

**Table:**

*D*—H···*A*	*D*—H	H···*A*	*D*···*A*	*D*—H···*A*
C25—H5···O1^i^	0.97 (3)	2.57 (3)	3.256 (2)	128 (2)
O8—H6···O1^i^	0.88 (3)	1.91 (3)	2.781 (2)	168 (3)

Symmetry codes: (i) *x*, *y*−1, *z*.
